# Evaluation of Enamel Acid Resistance and Whitening Effect of the CAP System

**DOI:** 10.3390/dj10090161

**Published:** 2022-08-30

**Authors:** Naoko Miki, Yasuo Miake, Shinji Shimoda, Hiroyuki Mishima

**Affiliations:** 1 Medical Corporation Precious One, 1-2-3 Kanda Jimbocho, Chiyoda-ku, Tokyo 101-0051, Japan; 2 Department of Anatomy, Tsurumi University School of Dental Medicine, 2-1-3 Tsurumi-ku, Yokohama 230-8501, Japan; 3 Department of Dental Engineering, Tsurumi University School of Dental Medicine, 2-1-3 Tsurumi-ku, Yokohama 230-8501, Japan

**Keywords:** α-TCP, fluoride, APF, CAP system

## Abstract

This study aimed to compare the effectiveness of a novel professional tooth-strengthening system and a conventional caries-prevention method that involved the use of high fluoride concentrations, to determine whether the system has a whitening effect. Bovine tooth-enamel samples were treated with fluoride gel (conventional APF method) or a mixture of citric acid gel, calcium phosphate (α-TCP), and fluoride gel, referred to as the CAP system; these treatments were performed to generate an acid-resistant layer on the enamel surface. For the evaluation of the acid resistance, a cyclic experiment, involving a 1-h remineralization and a 24-h acid treatment, was conducted thrice after the treatments. The height profiles were observed using a 3D-measuring laser microscope and the hardness was evaluated by Vickers hardness test. The morphological changes in the surface and cross-section of the enamel were observed by scanning electron microscopy. To evaluate the whitening effect, the enamel was ground until the color of the underlying dentin was recognizable; the CAP system was applied once, and the color change was measured using a color difference meter. As a result, it was confirmed that an acid-resistant layer was formed on the tooth surfaces treated with the CAP system, and a whitening effect was obtained.

## 1. Introduction

Dental caries are caused by the progression of demineralization due to acid contact with the hydroxyapatite in tooth enamel, but if the acid resistance of the tooth surface can be improved, the progression of demineralization can be inhibited and dental caries can be prevented [1,2]. Recently, prophylactic treatments aimed at improving the acid resistance of teeth, such as the application of casein phosphopeptide (CPP) to the tooth surface and the incorporation of nanoapatite into the toothpaste, have been investigated [3,4,5]. In addition, new caries-prevention methods involving various approaches are being developed, such as the use of bioactive peptides to inhibit *Streptococcus mutans* biofilm formation and bacterial attachment to tooth surfaces [6]. However, while multiple self-care approaches are being available, professional caries-prevention methods are currently limited to fluoride application [1]. All of the fluoride formulations currently used for topical application deposit soluble calcium fluoride on the tooth surface [7]. Calcium fluoride serves as a source of calcium for teeth that have lost calcium through demineralization and provides the raw material for the formation of fluorapatite [8]. α-Tricalcium phosphate (α-TCP) is one of the calcium phosphates represented by the molecular formula Ca_3_(PO_4_)_2_ and is a useful bioceramic material [9]. In addition to its use in artificial bones and bone fillers, it is widely applied in dental cements and toothpastes [9,10]. TCP is available in two forms: α-type for high temperature phase and β-type for low temperature phase. The α-type undergoes a phase transition to hydroxyapatite in environments with a pH higher than 7 [11]. We focused on the bioactivity of α-TCP, its transformation to hydroxyapatite, and the reactivity of fluoride with calcium, and we examined its ability to enhance the acid resistance of the teeth and whitening effect by simultaneously coating the teeth with fluoride gel and α-TCP (hereinafter referred to as the CAP system). The purpose of this study was to determine the effectiveness of the CAP system in enhancing enamel surface hardness and acid resistance, as compared to conventional caries-prevention methods that apply high concentrations of fluoride, and to determine the extent of the whitening effect of the CAP system [3,4,5,12,13].

## 2. Materials and Methods

### 2.1. Preparation of Enamel Samples

Twenty-five blocks of enamel on the labial side of the anterior crown of bovine teeth were prepared (approximately 10 mm × 10 mm × 5 mm) and used as samples. The enamel surface was mirror polished using a water-resistant abrasive paper (#800, 1200, 3200, 4000; Reflex NAC, PRESI) and each sample was polished for approximately 3 min. A 5 mm × 5 mm window was created using inlay wax. The area covered with wax was set as the reference surface.

### 2.2. Reagents

The following reagents were used in the experiment:

1. Calcium phosphate (a-powder): α-TCP, φ8–15 μm (Taihei Chemical Industrial Co. Ltd., Osaka, Japan);
2. Citric acid gel (C-gel): 5% citric acid, 0.01% malic acid, 5% glycerin, 3% tamarind gum, others (pH 3.0) (AIWA Co. Ltd. Osaka, Japan);
3. Fluoride gel (APF, Fluor Jelly^®^): 2% NaF, (pH 3.5) (Bee Brand Medico Dental Co. Ltd., Osaka, Japan);
4. Gel for neutralization (CR-gel): 1.0% Na5P3O10, 0.01% NaOH, 10% glycerin, 1.5% xanthan gum, 1.0% cellulose gum, others (pH 9.3) (AIWA, Co. Ltd. Osaka, Japan);
5. Remineralization solution (for acid challenge experiment): 0.02M HEPES (Ca: 3 mM, P: 1.8 mM, pH adjustment to 7.3 using KOH);
6. Demineralization solution (for acid challenge experiment): 0.1-M lactate buffer solution (2.5% hydroxyethyl cellulose) (Ca: 3 mM, P: 1.8 mM, pH adjustment: KOH; pH 4.5).

### 2.3. Acid Resistance Strengthening of Teeth

Five polished enamel blocks were used. After ionizing some of the crystals on the surface by applying citric acid gel (C-gel) to the surface, calcium phosphate (a-powder) and C-gel were mixed in a 1:2 weight ratio and applied to the surface. Fluoride (Fluor Jelly^®^) was then applied to deposit calcium phosphate on the tooth surface. Finally, an alkaline agent (CR-gel) was applied to neutralize and further crystallize the ionized calcium and phosphoric acid. The procedure, referred to as the CAP system, is outlined below:

1. C-gel application for 30 s;
2. Wipe with melamine foam;
3. Application of a-powder and C-gel mixture for 1 min (weight ratio 1:2);
4. Application of Fluor Jelly^®^ for 3 min;
5. Wipe with melamine foam;
6. Application of CR-gel for 1 min;
7. Washing with water.

### 2.4. Acid Challenge Experiment

The acid challenge experiments were performed to determine the acid resistance of the bovine tooth enamel samples. The following three experimental conditions were used: (1) no fluoride application (control group), (2) conventional acidulated phosphate fluoride (APF) method (9000 ppm F, at pH 3.6, applied for 4 min), and (3) CAP system. Five samples (*n* = 5) were used for each experimental group. After treatment, the specimens were immersed in 0.02-M HEPES remineralization solution (Ca: 3 mM, P: 1.8 mM, pH 7.3) at 37 °C for 1 h, followed by immersion in 0.1 M lactic acid demineralization solution (Ca: 3 mM, P: 1.8 mM, pH 4.5) at 37 °C for 24 h for a total of three cycles; these specimens were used for acid resistance measurement. During the demineralization and remineralization processes, five samples for each group were suspended by a thread in a 300-mL beaker and reacted with approximately 50 mL of the solution per sample.

### 2.5. Measurement of Height Difference Profiles

The samples were dehydrated in an ascending ethanol series after wax removal, and their height profiles were measured using a 3D measurement laser microscope (LEXT OLS4000; Olympus, Tokyo, Japan). An area of 645 μm × 645 μm, in the central region of the sample window boundary, was photographed at 20 × magnification. The surface that was covered with wax was used as the reference surface and the surface that was subjected to the acid challenge was used as the experimental surface, and the height difference between the reference and experimental surfaces was measured. The height difference was measured at five locations per sample, and the means ± standard deviations were calculated.

### 2.6. Micro Vickers Hardness Measurement

The Vickers hardness values were measured using a hardness tester after the samples were dehydrated in an ascending ethanol series (HMV-1; Shimadzu Corporation, Tokyo, Japan). The indentation load was set to 0.49 N and the time, to 20 s to measure the Vickers hardness (HV). To compensate for errors due to individual differences in the samples, the change in HV before and after the experiment (ΔHV = symmetry plane—experimental plane) was calculated. The HV and ΔHV values were measured at five locations per sample, and the means value ± standard deviations were calculated.

### 2.7. Scanning Electron Microscopy (SEM)

Gold or carbon deposition was performed on the surface of the samples that had undergone the CAP system treatment and acid challenge experiments using a vacuum deposition system (SC-701AT; Quick Auto Coater, Tokyo, Japan; VC-100S, Vacuum device; Ibaragi, Japan). The acceleration voltage of the scanning electron microscope (SU6600; Hitachi, Tokyo, Japan) (JSM-5600; JEOL, Tokyo, Japan) was set to 15 kV to obtain the SEM images. The samples were then embedded in Rigolac resin (Nissin EM, Tokyo, Japan), and cross sections were cut perpendicular to the surface; the changes in the acid-resistant layer and demineralization depth were observed on the SEM images.

### 2.8. Whitening Effect Experiment

Five enamel blocks were used in the experiment. The enamel was ground (thickness, <1 mm) until the color of the dentin became apparent. The L*a*b* values of the samples were measured using a color difference meter (VITA Easyshade^®^ V, Bad Sackingen, Germany). The CAP system was then applied to the samples, and the color tone changes were measured and compared. The measurements were recorded three times for each sample, and the average values were used in the analysis. The color change (ΔE) was calculated using the following equation:

ΔE = [(ΔL*)^2^ + (Δa*)^2^ + (Δb*)^2^]^1/2^

### 2.9. Statistical Processing

The result of the Vickers hardness experiment were subjected to one-way analysis of variance (ANOVA) to determine the statistical differences among the groups, with statistical significance set at *p* < 0.01. The data for each group are presented as means ± SDs of five replicates. The Bonferroni test was used for post-hoc comparisons when the analysis of variance was determined to be significant (*p* < 0.01). The analysis software Origin 2022 (2022, Lightstone Corp, Tokyo, Japan) was used.

## 3. Results

### 3.1. Observation of Enamel Surface

**Figure 1:**
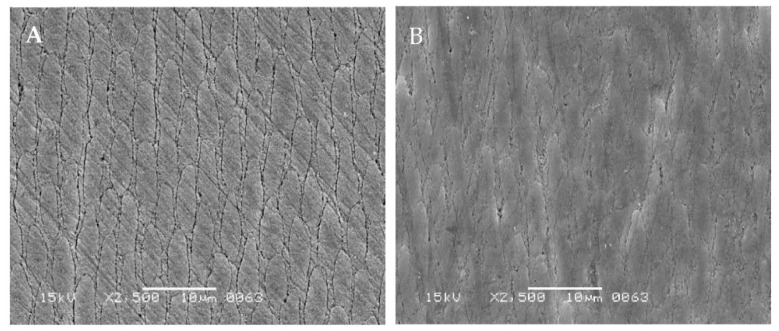
Scanning electron microscopy images of enamel surface. (**A**) C-gel-treated enamel surface; (**B**) Tooth surface after CAP system treatment. (Gold deposition sample, Scale bar, 10 μm).

A comparison of the CAP system-treated processes is shown. Specifically, the surface scanning electron microscopy (SEM) images of the enamel are shown in Figure 1. The C-gel-treated enamel surface showed a rough surface with a clear enamel sheath structure (Figure 1A). The tooth surfaces treated with the CAP system showed many areas where the enamel sheath structure was obscured and flattened (Figure 1B).

### 3.2. Height Difference Profiles 

**Figure 2:**
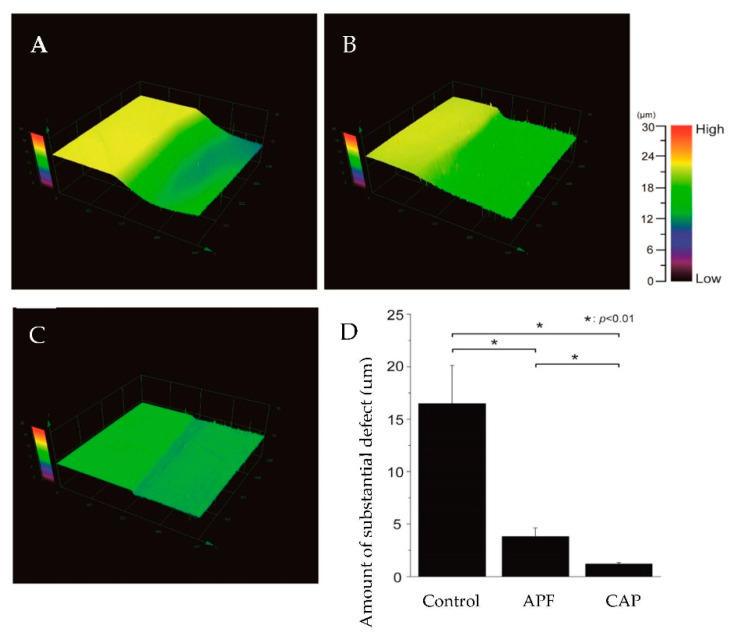
Height difference profiles measured using a 3D measurement laser microscope. (**A**) Boundary images of the reference and experimental surfaces after the acid challenge of the control; (**B**) APF; and (**C**) CAP system groups; (**D**) Graphical representation of substantial defects due to demineralization (*n* = 5) (* *p* < 0.01).

The images of 3D laser microscopy after the acid challenge and graphs of the measurement results are shown in Figure 2.

The left sides of Figure 2A–C show that the reference surface was protected by wax and not demineralized, while the right sides show the demineralization of the experimental surface. In the control group, the experimental surface was severely demineralized, with 16.481 ± 3.656-μm deep defects in the superficial enamel layer (Figure 2A,D). In the APF group (Figure 2B), the difference in the heights between the control and experimental surfaces decreased to 3.801 ± 0.844 μm, indicating a significant inhibition of the demineralization as compared to that in the control group (*p* < 0.01) (Figure 2B,D). The CAP system group, Figure 2C, showed a smaller difference in height than the APF group of 1.208 ± 0.117 μm, indicating a significant difference between the CAP system and APF groups (*p* < 0.01). The CAP system group had the smallest difference between the reference and experimental surfaces compared to the control and APF groups (*p* < 0.01) (Figure 2C,D).

### 3.3. Micro Vickers Hardness Measurement

**Figure 3:**
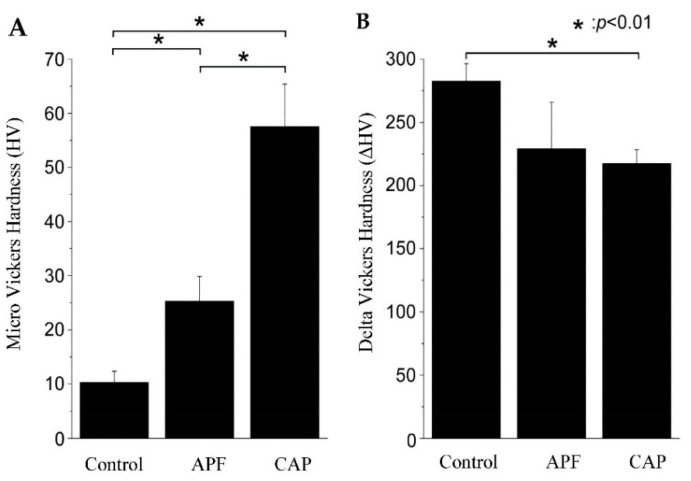
Vickers hardness measurements. (**A**) Graph of Vickers hardness (HV) values after acid challenge (*n* = 5) (* *p* < 0.01); (**B**) Graph of ΔHV values (difference in the HV values between the reference and experimental surfaces) values.

Figure 3 shows the HV and ΔHV values of the demineralized surface after the acid challenge. The control group had the lowest HV among the three groups at 10.29 ± 2.10, which was significantly smaller than the values reported for both the APF and CAP system groups (*p* < 0.01) (Figure 3A). The APF group had an HV value of 25.32 ± 4.55, which was an improvement from that of the control group. The CAP system group had the highest HV among the groups at 57.48 ± 7.88, which was significantly different from the values of the control and APF groups (*p* < 0.01) (Figure 3A). The ΔHV values were 263.33 ± 28.41 HV for the control group, 250.27 ± 39.17 HV for the APF group, and 235.12 ± 27.76 HV for the CAP system group. The CAP system group showed a significant decrease in the HV value compared to the control group (*p* < 0.01) (Figure 3B; Table 1)

### 3.4. SEM after Acid Challenge

**Figure 4:**
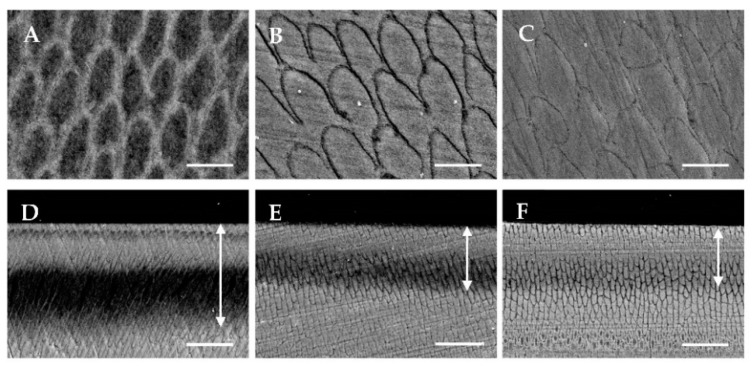
Scanning electron microscopy (SEM) images of enamel surface and cross-sections after acid challenge. (**A**) Surface SEM image of the control group; (**B**) APF group; and (**C**) CAP system group. (Carbon deposition sample, (**A**–**C**): Scale bar is 5.0 μm); (**D**) Cross-sectional SEM image of the control group; (**E**) APF group; and (**F**) CAP system group. (Carbon deposition sample, (**D**–**F**): Scale bar is 25 μm; arrows indicate demineralization depth).

The surface and cross-sectional SEM images of the enamel surface after the acid challenge are shown in Figure 4. The SEM image of the surface of the control group showed that the acid challenge dissolved the enamel columns, resulting in a rough appearance due to demineralization (Figure 4A). In the APF group, the dissolution of the enamel rod was decreased, but the demineralization of the enamel sheath was apparent (Figure 4B). The surface of the CAP system group had a nearly flat image, and the unevenness of the enamel sheath, as seen in the APF group, was decreased (Figure 4C).

The cross-sectional SEM images of the control group showed demineralization; the columnar structures were obscured at a depth of 20–40 μm (arrow) below the surface; the parenchymal defects were also visualized (Figure 4D). The APF group showed sub-surface demineralization at 20–30 μm (arrow) from the surface, but the columnar structure had not collapsed, and an enlarged enamel sheath was observed (Figure 4E). The CAP system group also showed sub-surface demineralization at about 30 μm (arrow) below the surface, but to a lesser extent than the APF group, and the enamel sheath was less enlarged (Figure 4F). Compared to the APF group, the CAP system group had a higher signal intensity in the superficial layers and in the layers deeper than 30 μm. Furthermore, the signal intensity in the demineralized layer was the highest in the CAP system group when compared among the three groups (Figure 4D–F).

### 3.5. Whitening Effect Experiment

**Figure 5:**
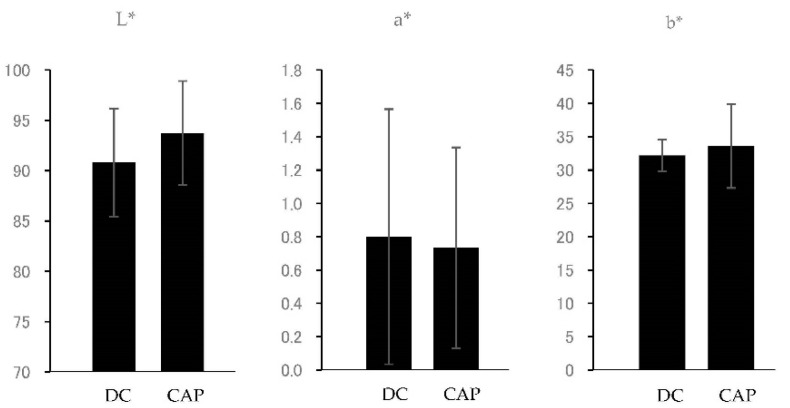
Graphs showing the L*a*b* values. DC, dentin color; CAP, CAP system treatment.

The results of the whitening effect are shown in Figure 5 and Table 2. In the CAP system samples, the enamel tones showed an increase with a significant difference in L* values (*p* < 0.05), indicating an increase in brightness. For the a* and b* values, the a* values showed a decreasing trend and b* values showed an increasing trend, but the differences were not significant. The value of ΔE was 6.83 ± 3.13, indicating that a change in color difference was occurring. Therefore, the entire tooth surface was brightened in conjunction with the increase in L*, indicating that the dentin color was suppressed.

## 4. Discussion

The presence of flat, acid-resistant layers (Figure 1B) observed in the SEM images of the enamel surface layer treated with the CAP system suggests that a calcium phosphate coating was formed by the combination of α-TCP and fluoride (Figure 1). This acid-resistant layer is thought to be the result of the ionization of large numbers of calcium and phosphate ions in α-TCP brought about by citric acid; these ions react with fluoride ions to precipitate as calcium fluoride and fluorapatite, which also binds to the undissolved α-TCP. Fluorine is said to cause crystal nucleation and growth in the transformation of apatite, and some of the crystals deposited by the fluorine application and neutralization processes are thought to be deposited as hydroxyapatite and to be chemically bound to the enamel crystals [12,13].

Several methods of applying calcium solutions to the enamel surface layer to form a coating have been reported in previous studies [6,14,15]. Soares et al. analyzed tooth mineralization by micro-energy dispersive X-ray fluorescence; they applied APF gel containing nanohydroxyapatite (nHAp) to the enamel and found that the enamel in the nHAP-treated group had higher levels of calcium and phosphorus than the enamel in the APF group [14]. Similarly, in our study, the CAP system group showed a decline in the amount of parenchymatous defects in the teeth and maintained higher HV values than the APF group (Figure 2 and Figure 3). The previous studies have also shown that when the teeth are demineralized, their microstructure changes and hardness decreases [16]; HV is used for the qualitative evaluation of the degree of enamel demineralization and is considered an indicator of tooth acid resistance in the fields of dental engineering and conservative dentistry [17,18].

One of the objectives of this study was to determine whether there was a difference in the acid resistance of the CAP system compared to that of the conventional APF application method. The 3D laser microscopy step profiles revealed the depth of parenchymal defects, with the control group having the deepest defects, followed by the APF group, and the CAP system group had the shallowest parenchymal defects (Figure 2). Therefore, the CAP System treatment was shown to be more acid resistant than the conventional APF tooth-surface application method.

In this study, the HV of the CAP system group after the acid challenge was 57.48 ± 7.88, which was the highest among the three groups and about twice that of the APF group (Figure 3A). Even the ΔHV, corrected for errors due to the individual differences in the samples, showed a clear and significant difference between the control and after the CAP system treatment. This indicates that the CAP system treatment suppressed the decrease in hardness. This inhibition may also be related to the maintenance of the enamel structure. This was because the cross-sectional SEM observations after the acid loading showed that the enamel sheath expansion was suppressed and the structure was not destroyed in the CAP system group (Figure 4F). The SEM images of the CAP system group after the acid challenge showed no significant demineralization of the tooth surface (Figure 4C). Thus, the acid-resistant layer obtained by the CAP system may have provided a source of calcium and fluoride ions to the enamel during the demineralization test. In Figure 4A,B, the locations are shown where the demineralization progresses are different. Such differences are common in enamel caries and are thought to be due to differences in the degree of partial calcification. In this study, the presence or absence of fluoride may have had an effect, but the details are unknown. The previous studies on the application of hydroxyapatite on enamel have shown that nano-hydroxyapatite supplies calcium to the oral cavity, eventually decreasing the level of demineralization [19]. Furthermore, calcium phosphate and hydroxyapatite have been reported to function as calcium reservoirs during dissolution, which may promote the supersaturation of the enamel minerals, inhibit demineralization, and enhance remineralization [20,21,22]. The usefulness of maintaining calcium and fluoride reservoirs in the oral cavity is evident from the fact that toothpastes containing hydroxyapatite inhibit tooth demineralization [8,23,24]. The tooth surface application in this study was performed under the professional care of a dentist, and the results suggest that it effectively enhances the demineralization inhibitory effect in an environment with a high fluoride concentration.

Regarding the whitening effect, in the preliminary experiments, the samples with stained surfaces showed a considerable whitening effect during the initial stage of the CAP system, i.e., C-gel use. The reason for this was thought to be that superficial staining was removed by the citric acid in the C-gel. Therefore, in this experiment, the samples were prepared under the assumption that the enamel was thinned and the dentin color affected tooth color. The results showed an increase in enamel color tone, indicating an increase in lightness and whiteness. For the a* and b* value, the differences were not significant. The ΔE value indicated that a change in color difference was occurring. Thus, the overall brightening together with the increase in L* indicated that the dentin color was suppressed. This change in color tone is attributed not only to the white color of the applied calcium phosphate but also to the light-scattering phenomenon caused by the disorder of the crystal arrangement due to remineralization on the surface and inside the enamel. In this study, the operating procedures were shortened in consideration of the chair time in clinical settings. However, it is highly likely that extending the application time of fluoride and neutralizers is effective in preventing dental caries and acid erosion, enhancing the whitening effect, and preventing root surface hypersensitivity.

## 5. Conclusions

The formation of an acid-resistant layer of enamel was observed with the application of a new tooth-strengthening method (CAP system) using α-TCP and high concentrations of fluoride. The acid challenge experiments on bovine tooth enamel showed that the CAP system group decreased the number of parenchymatous defects in the teeth and improved the acid resistance more than the APF application group. Therefore, the CAP system is a more effective caries-preventive method than the APF tooth-surface application method currently used in clinical settings, and it also has a whitening effect.

## Figures and Tables

**Table 1:** Results of the measurement of HV and ΔHV values.

	Cont	APF	CAP
HV	10.29 ± 2.10	25.32 ± 4.55	57.48 ± 7.88
ΔHV	263.334 ± 28.407	250.27 ± 39.169	235.12 ± 27.759

**Table 2:** Results of the measurement of L*a*b* values.

	L*	a*	b*	ΔE
DC	90.81 ± 5.37	0.80 ± 0.77	32.17 ± 2.40	6.83 ± 3.13
CAP	93.74 ± 5.18	0.73 ± 0.60	33.60 ± 6.29	

DC, dentin color; CAP, CAP system treatment.

## Data Availability

Not applicable.
